# DNA-methylation age and accelerated epigenetic aging in blood as a tumor marker for predicting breast cancer susceptibility

**DOI:** 10.18632/aging.206169

**Published:** 2024-12-05

**Authors:** Su Yon Jung, Herbert Yu, Youping Deng, Matteo Pellegrini

**Affiliations:** 1 Translational Sciences Section, School of Nursing, University of California, Los Angeles, CA 90095, USA; 2 Department of Epidemiology, Fielding School of Public Health, University of California, Los Angeles, CA 90095, USA; 3 Jonsson Comprehensive Cancer Center, University of California, Los Angeles, CA 90095, USA; 4 Cancer Epidemiology Program, University of Hawaii Cancer Center, Honolulu, HI 96813, USA; 5 Department of Quantitative Health Sciences, Bioinformatics Core, John A. Burns School of Medicine, University of Hawaii Cancer Center, Honolulu, HI 96813, USA; 6 Department of Molecular, Cell and Developmental Biology, Life Sciences Division, University of California, Los Angeles, CA 90095, USA

**Keywords:** DNA methylation-based marker of aging, pre-diagnostic DNA, breast cancer, tumorigenesis, postmenopausal women

## Abstract

Background: DNA methylation (DNAm)–based marker of aging, referred to as ‘epigenetic age’ or ‘DNAm age’ is a highly accurate multi-tissue biomarker for aging, associated with age-related disease risk, including cancer. Breast cancer (BC), an age-associated disease, is associated with older DNAm age and epigenetic age acceleration (age accel) at tissue levels. But this raises a question on the predictability of DNAm age/age accel in BC development, emphasizing the importance of studying DNAm age in pre-diagnostic peripheral blood (PB) in BC etiology and prevention.

Methods: We included postmenopausal women from the largest study cohort and prospectively investigated BC development with their pre-diagnostic DNAm in PB leukocytes (PBLs). We estimated Horvath’s pan-tissue DNAm age and investigated whether DNAm age/age accel highly correlates with risk for developing subtype-specific BC and to what degree the risk is modified by hormones and lifestyle factors.

Results: DNAm age in PBLs was tightly correlated with age in this age range, and older DNAm age and epigenetic age accel were significantly associated with risk for developing overall BC and luminal subtypes. Of note, in women with bilateral oophorectomy before natural menopause experiencing shorter lifetime estrogen exposure than those with natural menopause, epigenetic age accel substantially influenced BC development, independent of obesity status and exogeneous estrogen use.

Conclusions: Our findings contribute to better understanding of biologic aging processes that mediate BC carcinogenesis, detecting a non-invasive epigenetic aging marker that better reflects BC development, and ultimately identifying the elderly with high risk who can benefit from epigenetically targeted preventive interventions.

## INTRODUCTION

Chronologic age is a well-established risk factor for chronic diseases, cancers, and death [1, 2]. However, individuals of the same chronologic age may accumulate biologic changes at different rates, so age may not truly reflect individuals’ time-related biologic alterations [3]. Increasing age correlates with various molecular alterations, including genetic changes associated with the deregulation of cellular processes and genomic instability, but differences in the biologic aging process at the individual level may be only partially explained by genetic mutations [4]. The DNA methylation (DNAm)–based marker of aging, referred to as ‘epigenetic age’ or ‘DNAm age’ has been known to capture well both the influences of genetic and environmental factors and their interplay across time in cellular functions; thus, it is a highly accurate multi-tissue biomarker of aging, strongly correlated with chronologic age in different tissues [5–8]. DNAm age in various tissues/organs has in turn been found to be associated with all-cause mortality [9–13], frailty [14], obesity [15], and diseases [16–23], including cancers [24–27].

Breast cancer (BC) is an age-associated disease with a sharp increase in incidence after menopause [28, 29]. DNAm age estimated in both normal breast tissues and paired peripheral blood has a strong linear relationship with chronologic age [30], and the two measures correlate well with each other [12, 30]. Further, increasing DNAm age and epigenetic age acceleration (age accel, defined as DNAm age that exceeds chronologic age) have been observed in BC tissues compared with normal and adjacent normal breast tissues [31]. A gradual accumulation of DNAm changes may occur through stochastic events, resulting in clonal expansion of stem or progenitor cells, contributing to the age-associated increase in the risk of developing BC [32–34]. However, the DNAm patterns in tumor tissues differ from those in normal tissues, exhibiting stem cells with the lowest DNAm age [6]. Thus, the tumor tissue–based DNAm age may indicate only the state of aging in tumor cells [35], which is modified by cancer cells’ capability of differentiation in malignant clones, raising the question of whether DNAm age/age accel in BC tissues can explain the cause and consequence of BC development.

A study of DNAm age in peripheral blood (PB) to prospectively investigate BC development is important in BC etiology and cancer prevention. A few studies [26, 36, 37] have tested for the association of DNAm age in PB with prospective development of BC, but with limited effect sizes for BC risk. Indeed, BC is highly heterogeneous at both the histologic and molecular levels, characterized by different molecular subtypes [29]. One premise for the inconclusive results from the prior studies is partly because they did not consider BC subtypes [26, 37] or analyzed them with insufficient statistic power [36]. BC is also a hormone-derived cancer. Of particular note, the effect of female hormones on biologic aging differs according to menopausal status. Before menopause, the hormones play a major role in accelerating breast tissue aging [38–40], and this epigenetic age accel is an important etiologic element for BC in pre-menopausal women [36]. However, the relationship between biologic aging and hormones is not straightforward in post-menopausal women. Some studies have reported higher epigenetic age accel associated with earlier age at menarche [8, 40], indicating that longer lifetime exposure to estrogen drives breast tissue–specific aging. In contrast, earlier menopause and a longer time since menopause were associated with epigenetic age accel in blood and tissues [22], reflecting that faster reproductive aging contributes to higher epigenetic age. Moreover, in addition to this endogenous hormonal factor as a putative marker for DNAm age, other conventional BC risk factors, such as obesity, alcohol, smoking, and exogenous hormone therapy [39, 41–46], can also affect epigenetic aging in postmenopausal women, modifying the risk for BC development. Studying both putative and established BC risk factors in relation to DNAm age and epigenetic age accel may provide deeper insight into BC carcinogenesis involving these factors and how they mediate the risk through the epigenetic aging process.

Our study focused on post-menopausal women, a population vulnerable to a high incidence of BC with complex hormonal effects on the biologic aging process. We prospectively investigated their BC development and examined their hormonal and lifestyle factors in association with DNAm age in PB leukocytes (PBLs), an easily accessible biofluid. We further investigated whether the blood-based DNAm age highly correlates with prospective development of BC in a BC subtype–specific manner and to what degree the risk prediction is modified by hormonal and lifestyle factors. Our study seeks to detect a non-invasive epigenetic aging marker that may better predict risk for BC development, contributing to identifying a risk group who can benefit from epigenetically informed preventive interventions.

## MATERIALS AND METHODS

### Study population

We used data from the Women’s Health Initiative Database for Genotypes and Phenotypes (WHI-dbGaP) genetic repository, which is derived from the largest prospective cohort study of postmenopausal women who had been enrolled at 50–79 years between 1993 and 1998 at more than 40 U.S. clinical centers [47, 48]. From the WHI-dbGaP, we obtained the genome-wide DNAm data in PBLs available in the BAA23, the largest ancillary study (AS), by repurposing the data for our study [49]. Because DNAm age has different patterns in different races [50], our study focused on only non–Hispanic white women, a majority of the AS population. Of 2,107 total, 998 whites were intially included. After removing women who had been diagnosed with any cancers at enrollment and/or were followed for less than 1 year, we finally included 956 women, 66 of whom developed primary invasive BC during a 17-year mean follow-up.

For validation analysis, we used independent data with global-level DNAm in PBLs from the National Center for Biotechnology Information Gene Expression Omnibus (GEO) database. This data (accession numbers of GSE51032) was generated by the European Prospective Investigation into Cancer and Nutrition (EPIC-Italy) from the Human Genetics Foundation in Turin, Italy [37, 51], containing 233 women who prospectively developed primary BC and 340 women who remained cancer-free. The institutional review boards of each WHI clinical center and the University of California, Los Angeles, approved this study.

### Data collection and BC outcomes

Women enrolled in the WHI completed self-administered questionnaires at screening and provided demographic information (e.g., age and race/ethnicity), comorbid conditions (e.g., ever having been treated for diabetes [DM]), lifestyle factors (e.g., daily diet, including dietary alcohol intake; Healthy Eating Index [HEI]-2015; and pack-years of smoking), and reproductive histories (e.g., a history of oophorectomy; durations of two types of exogenous estrogen [E] use, including unopposed E-only and opposed E plus progestin [P] from pills or patches). Trained staff acquired anthropometric measurements, including height, weight, and waist and hip circumferences at baseline. Primary invasive BC development among the WHI participants was determined by a committee of physicians through a review of the patients’ medical records and pathology reports, and then coded according to the National Cancer Institute’s Surveillance, Epidemiology, and End-Results guidelines [52]. The time from enrollment to BC development, censoring, or study end-point (by March 6, 2021) was calculated as numbers of years. Global-DNAm data in PBLs obtained from the GEO database included participants’ sex, age, and primary BC development by follow-up to 2010.

### Genome-wide DNAm array and epigenetic clock of aging

DNAm array at the global level for the WHI participants was performed using their PBL DNA samples extracted at baseline, via Illumina 450 BeadChip. Basic quality control (QC) was performed by excluding cross-reactive probes and probes with a detection *p* > 0.01 (poor performance) in more than 10% of samples to avoid spurious associations. DNAm data were beta-mixture quantile (BMIQ)-normalized [53] and batched-adjusted with random intercept for plate and chip and a fixed effect for row [54], resulting in 482,421 CpG dinucleotides (CpGs). For DNAm stability measured from stored samples [55], we followed Horvath’s suggestion [6], estimating leukocyte heterogeneities to be controlled for in generating DNAm age by using Houseman’s method [56] (for CD4^+^ T cells, natural killer cells, monocytes, and granulocytes) and Horvath’s method [6] (for plasma blasts, CD8^+^CD28^–^CD45RA^–^ T cells, and naïve CD8 T cells).

Genome-wide DNAm in PBLs of GSE51032 was generated via Illumina 450 BeadChip, and after similar basic QC, the data were normalized via background correction based on normal-exponential out-of-band (Noob) [57] using *minfi*, resulting in 485,512 CpGs. Leukocyte heterogeneities were also estimated for generating DNAm age independent of different cell counts.

The epigenetic clock of aging is a prediction of chronologic age based on the individual’s DNAm levels. We used Horvath’s method [6, 58], a pan-tissue predictor on the basis of 353 selected CpGs, which is the most popular well-established epigenetic age estimator. DNAm age is a composite scale by a linear combination of the weighted CpGs. This was generated via an available online tool [6, 58] and the *methylclock* annotation Bioconductor package. The departure of epigenetic age from chronologic age was investigated by two estimates: 1) ‘AgeAccelDiff’ defined by departure of DNAm age from chronologic age measured by subtracting chronologic age from DNAm age; and 2) ‘intrinsic epigenetic age acceleration (IEAA)’, defined as the residual from regressing DNAm age on chronologic age, which accounts for blood cell proportions. The IEAA reflects cell-intrinsic aging effects, independent of variations of DNAm levels owing to heterogeneity in cell components between individuals [59].

### Statistical analysis

For the correlation of DNAm age and two epigenetic age-departure estimates (AgeAccelDiff and IEAA) with chronologic age, we performed linear regression and Spearman’s correlation overall and by BC status and selective reproductive histories. Differences in levels of DNAm age and the two age-departure measures by conventional BC risk factors were tested using unpaired two-sample t- or one-way ANOVA tests when applicable. If continuous variables were skewed or had outliers, Mann-Whitney/Wilcoxon’s rank-sum and Kruskal-Wallis tests were used as appropriate. DNAm age and the two age-departure measures were further regressed on individual risk factors, referring to a one-unit increase in the risk factor associated with increase in DNAm age in units of years.

The distributions of DNAm age and the two age-departure estimates in overall BC and by BC subtype were examined via unpaired two-sample t- or Mann-Whitney test as appropriate. In particular, we dichotomously categorized the two age-departure measures into age accel and age deceleration (age decel) and performed the Kaplan-Meier curve and a log rank test. We conducted a multiple Cox proportional hazards regression for association between DNAm age/age departure and BC development, with an assumption test met via a Schoenfeld residual plot and rho, adjusting for conventional BC risk factors [39, 41–46], including body mass index (BMI), waist-to-hip ratio (WHR), DM, HEI-2015, alcohol intake, smoking, a history of oophorectomy, and exogenous hormone use. Hazard ratio (HR) from the analysis reflects a one-year older DNAm age and age accel increase risk for BC development. DNAm age and age accel were further analyzed as a 10-year interval with different segments of the follow-up period. Given that the testing was performed on the basis of our hypothesis-driven questions (i.e., DNAm age associated with BC), a two-tailed *p* < 0.05 was considered significant.

We further conducted subset analyses by stratifying the study population between natural and artificial menopause (after bilateral oophorectomy) and tested for a multiplicative interaction to formally detect whether the effect of DNAm age and age accel on BC development is modified by the gynecologic surgery.

## RESULTS

### Correlation of DNAm age and epigenetic age-departure estimates (AgeAccelDiff and IEAA) with chronologic age (hereafter, age) and with conventional BC risk factors

**Figure 1:**
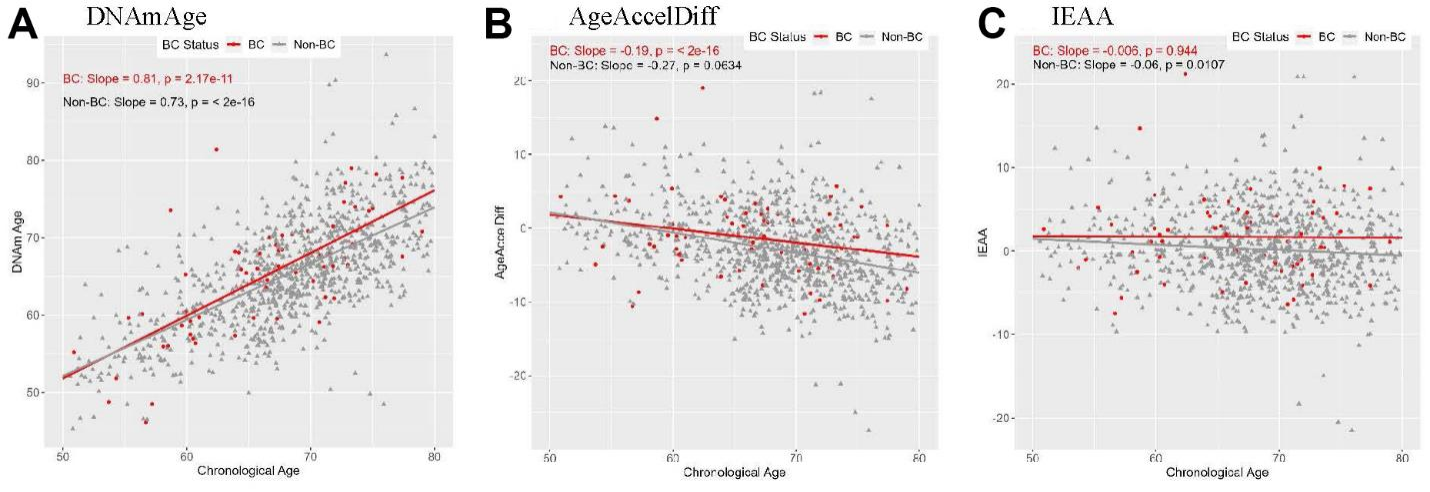
**Correlation between DNAmAge/AgeAccelDiff/IEAA and chronologic age by BC status**. (AgeAccelDiff, epigenetic age acceleration measured as departure of DNAmAge from chronologic age; IEAA, intrinsic epigenetic age acceleration measured as residuals by regressing DNAmAge on chronologic age, adjusted for cell composition; BC, breast cancer; DNAmAge, DNA methylation–based marker of aging). (**A**) DNAmAge (**B**) AgeAccelDiff (**C**) IEAA.

In both prospectively developed BC (hereafter, BC) and non-BC subgroups, DNAm age highly corrected with age, with slightly stronger correlation in the BC group (Figure 1). An epigenetic age accel (positive departure of DNAm from age) measured in AgeAccelDiff and IEAA was constant from 50 through 60 years in both groups, and in the BC group, the age accel did not much differ in older ages. Similar patterns were observed in women with intact ovaries and in those with no use of exogeneous E (Supplementary Figure 1). However, in women with bilateral oophorectomy who further developed BC, the age accel measured in IEAA increased with older age, despite insufficient statistical power. Consequently, in all women combined, those with bilateral oophorectomy had older DNAm age and increased age accel than those with both intact ovaries, although the results were not statistically significant (Supplementary Figure 2).

Exogeneous E users showed complex patterns that differed by the type of E used, length of the use, and BC status. Among unopposed E-only long-term (≥ 5 years) users, a slight degree of age decel (DNAm that falls behind age) in AgeAccelDiff and increased age accel in IEAA with older ages were observed in the BC group, whereas greater age decel in both measures with older ages were observed in non-BC group (Supplementary Figure 1G–1O). In all women combined, older DNAm age and increased age accel in AgeAccelDiff and IEAA were observed in E users than nonusers, with a nonlinear relationship among users of different durations, i.e., older DNAm and higher age accel in short-term (< 5 years) than in longer-term (≥ 5 years) users (Supplementary Figure 2M–2O). These short-term users had about a 2-year-older DNAm age than nonusers among combined and within non-BC women, but that was not observed in BC women (Table 1 and Supplementary Table 1).

**Table 1:** Association of DNAmAge with selected BC-risk factors*.

**BC risk factor**	**Effect size**	**95% CI**	***P***
Age at enrollment	**0.73**	**(0.68, 0.78)**	**1.51E-12**
Waist-to-hip ratio	**7.48**	**(1.93, 13.03)**	**0.008**
Waist-to-hip ratio** (≤ 0.85 vs. > 0.85)	**0.99**	**(0.10, 1.88)**	**0.029**
Healthy eating index-2015	**0.07**	**(0.03, 0.11)**	**0.001**
Healthy eating index-2015¥ (≤ 65.29 vs. > 65.29)	**1.52**	**(0.66, 2.38)**	**0.001**
Pack-years of smoking (never vs. < 5 years)	-0.21	(-1.66, 1.23)	0.771
5 to < 20 years	**-1.46**	**(-2.90, -0.02)**	**0.046**
20 + years	**-1.42**	**(-2.50, -0.34)**	**0.010**
Exogenous estrogen only (never use vs. < 5 years)	**1.95**	**(0.79, 3.11)**	**0.001**
5 to < 10 years	-1.08	(-3.12, 0.97)	0.302
10 + years	**1.92**	**(0.18, 3.66)**	**0.030**
Exogenous estrogen plus progestin (never use vs. < 5 years)	**-3.16**	**(-5.00, -1.33)**	**0.001**
5 to < 10 years	-1.78	(-5.36, 1.81)	0.331
10 + years	-2.90	(-6.94, 1.14)	0.159

BC, breast cancer; CI, confidence interval; DNAmAge, DNA methylation–based marker of aging. Numbers in bold face are statistically significant.

* Only factors having *statistically significant* association with DNAmAge are displayed.

** Waist-to-hip ratio was categorized using 0.85, where cutoff levels or higher fall within visceral obese range [60].

¥ Healthy eating index-2015 variable is dichotomized by a median (= 65.29).

However, users of opposed E plus P showed different patterns (Supplementary Figures 1P–1X, 2P–2R). In shorter-term (< 5 years) users, whereas a stronger correlation of DNAm age with age and increased age accel in both AgeAccelDiff and IEAA with older ages were present in the BC group, a less-strong correlation of DNAm age with age and more profound age decel with older ages were observed in the non-BC group. In overall and non-BC groups, these short-term users were associated with about a 3-year-younger DNAm age than nonusers (Table 1 and Supplementary Table 1).

**Figure 2:**
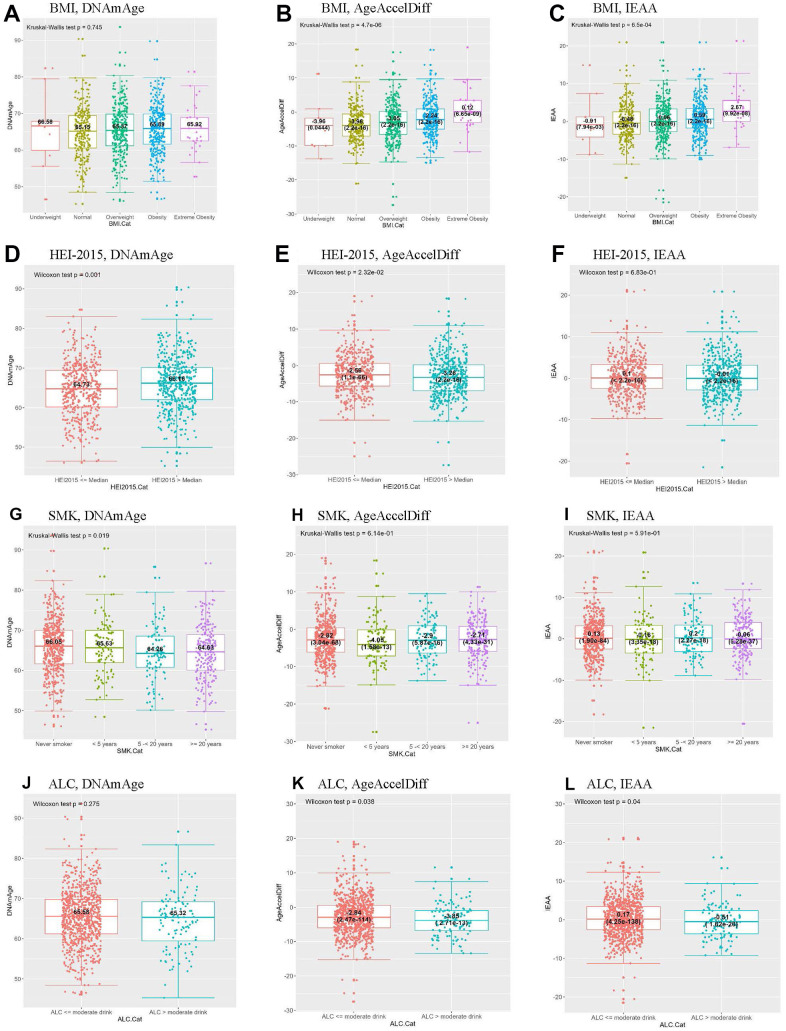
**Distribution of DNAmAge/AgeAccelDiff/IEAA by selected BC-risk factors.** By BMI, (**A**–**C**); HEI-2015, (**D**–**F**); SMK, (**G**–**I**); ALC, (**J**–**L**). (ALC, dietary alcohol categorized by moderate drink (14 g); AgeAccelDiff, epigenetic age acceleration as departure of DNAmAge from chronologic age; IEAA, intrinsic epigenetic age acceleration as residuals adjusted for cell composition; BC, breast cancer; BMI, body mass index; Cat, Categories; DNAmAge, DNA methylation–based marker of aging; HEI, healthy eating index; SMK, pack-years of smoking.) (**A**) BMI, DNAmAge (**B**) BMI, AgeAccelDiff (**C**) BMI, IEAA (**D**) HEI-2015, DNAmAge (**E**) HEI-2015, AgeAccelDiff (**F**) HEI-2015, IEAA (**G**) SMK, DNAmAge (**H**) SMK, AgeAccelDiff (**I**) SMK, IEAA (**J**) ALC, DNAmAge (**K**) ALC, AgeAccelDiff (**L**) ALC, IEAA.

BMI, waist, and WHR had a dose-response relationship with DNAm age and age accel (Figure 2 and Supplementary Figure 2). In particular, compared with women with normal BMI, extremely obese women (BMI > 40) had a 4-year increased age accel in AgeAccelDiff and IEAA in the women overall. Of note, the extremely obese women who developed BC had a 20-year increase in age accel by both measures and a 16-year-older DNAm age compared with those with normal BMI. Additionally, in both overall and non-BC groups, a 1-unit increase in WHR yielded a 4-year increase in age accel and about a 7-year-older DNAm age (Tables 1, 2 and Supplementary Tables 1–3).

**Table 2:** Association of epigenetic age acceleration with selected BC-risk factors*. A. AgeAccelDiff

**BC risk factor**	**Effect size**	**95% CI**	***P***
Age at enrollment	**-0.27**	**(-0.32, -0.22)**	**9.27E-24**
Body mass index, kg/m^2^	**0.14**	**(0.08, 0.20)**	**4.59E-06**
Body mass index, kg/m^2^ (≥ 18.5 to < 25, normal, vs. < 18.5, underweight)	-0.63	(-3.96**,** 2.70)	0.712
≥ 25 to < 30, overweight	0.56	(-0.30**,** 1.41)	0.205
≥ 30 to < 40, obesity	**1.70**	**(0.84, 2.56)**	**0.0001**
≥ 40, extreme obesity	**4.41**	**(2.44, 6.37)**	**1.19E-05**
Waist circumference, cm	**0.06**	**(0.03, 0.08)**	**2.85E-06**
Waist circumference, cm** (≤ 88 vs. > 88)	**1.07**	**(0.39, 1.75)**	**0.002**
Healthy eating index-2015	**-0.03**	**(-0.07, -0.002)**	**0.040**
Dietary alcohol per day, g	**-0.03**	**(-0.07, -0.001)**	**0.042**
Dietary alcohol per day, g¥ (≤ 14 vs. > 14)	**-1.04**	**(-2.07, -0.02)**	**0.045**
**B. IEAA**			
**BC risk factor**	**Effect size**	**95% CI**	***P***
Age at enrollment	**-0.07**	**(-0.11, -0.02)**	**0.009**
Body mass index, kg/m^2^	**0.10**	**(0.04, 0.15)**	**0.000**
Body mass index, kg/m^2^ (≥ 18.5 to < 25, normal, vs. < 18.5, underweight)	0.15	(-2.86, 3.16)	0.924
≥ 25 to < 30, overweight	0.48	(-0.30, 1.26)	0.224
≥ 30 to < 40, obesity	**1.12**	**(0.34, 1.89)**	**0.005**
≥ 40, extreme obesity	**3.47**	**(1.69, 5.24)**	**0.000**
Waist circumference, cm	**0.04**	**(0.02, 0.06)**	**0.000**
Waist circumference, cm** (≤ 88 vs. > 88)	**0.76**	**(0.15, 1.37)**	**0.014**
Waist-to-hip ratio	**4.06**	**(0.15, 7.97)**	**0.042**

AgeAccelDiff, epigenetic age acceleration measured as departure of DNAmAge from chronologic age; BC, breast cancer; CI, confidence interval; DNAmAge, DNA methylation–based marker of aging; IEAA, intrinsic epigenetic age acceleration as residuals by regressing DNAmAge on chronologic age, adjusted for cell composition. Numbers in bold face are statistically significant.

* Only factors having *statistically significant* association with DNAmAge are displayed.

** Waist circumference was categorized using 88 cm, where cutoff levels or higher fall within visceral obese range [60].

¥ Dietary alcohol per day is dichotomized by a moderate drink amount (= 14 g).

Analyses of alcohol intake and smoking showed the opposite patterns (Tables 1, 2 and Supplementary Table 1 and Figure 2). Compared with never smokers and moderate alcohol users (≤ 14 g/day in women), regular smokers for ≥ 5 years and > 14-g alcohol users had a 1.5-year-younger DNAm age and about a 1-year age decel in AgeAccelDiff, respectively. In addition, women with a higher score on the HEI-2015 scale, indicating better eating behaviors, had an older DNAm age than their counterparts, but they showed an age decel pattern in AgeAccelDiff. However, none of these risk factors substantially influenced epigenetic aging in the BC group.

### DNAm age and epigenetic age-departure with prospective development of BC

**Figure 3:**
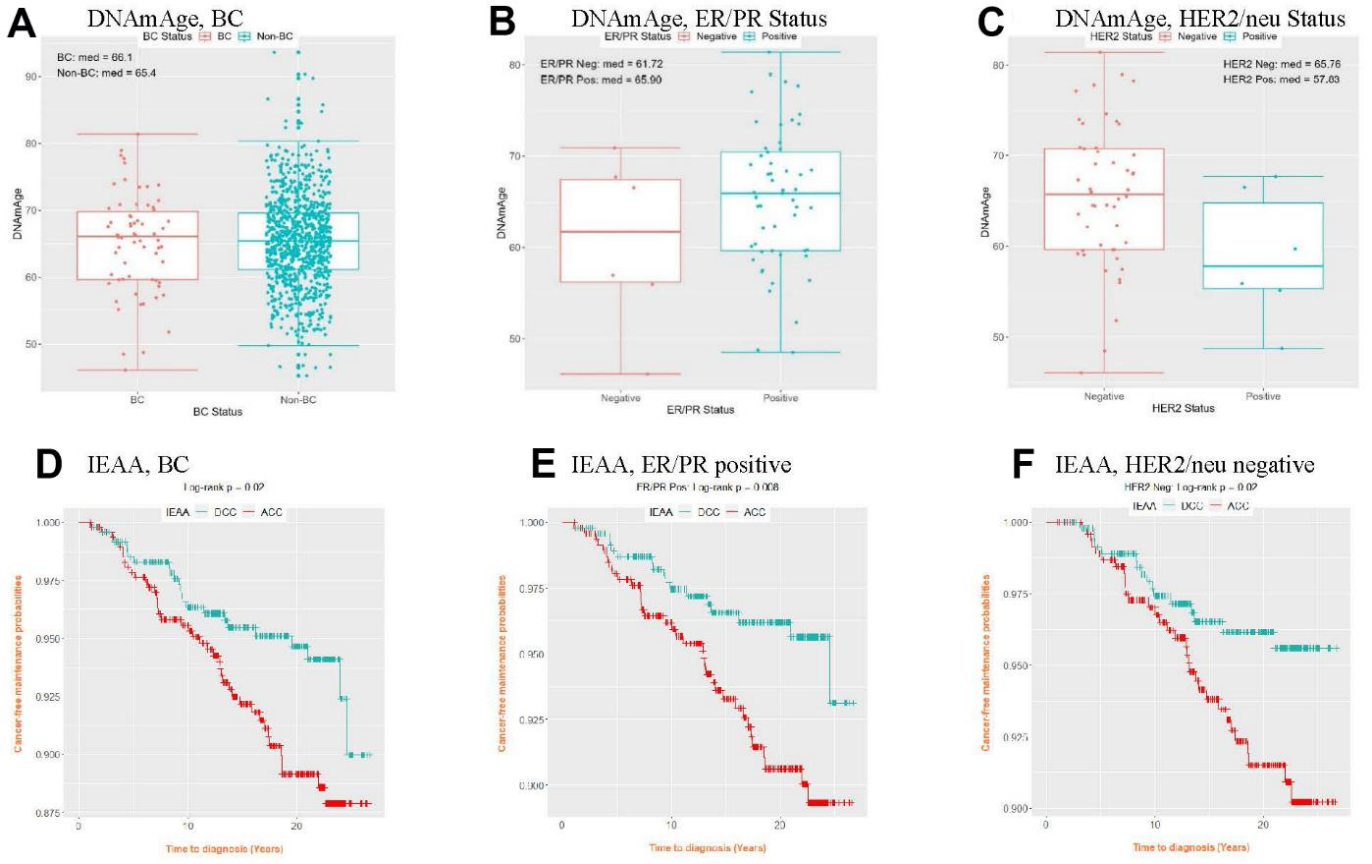
Distribution of DNAmAge (**A**–**C**) and cancer-free probability curve of IEAA (**D**–**F**) by BC status and BC subtype. (IEAA, intrinsic epigenetic age acceleration as residuals adjusted for cell composition; ACC, acceleration, i.e., positive residuals; BC, breast cancer; DCC, deceleration, i.e., negative residuals; DNAmAge, DNA methylation–based marker of aging; ER/PR, estrogen and progesterone receptor; HER2/neu, human epidermal growth factor receptor 2). (**A**) DNAmAge, BC (**B**) DNAmAge, ER/PR Status (**C**) DNAmAge, HER2/neu Status (**D**) IEAA, BC (**E**) IEAA, ER/PR positive (**F**) IEAA, HER2/neu negative.

Older DNAm age and greater age accel were observed in the BC group than in the non-BC group (Figure 3 and Supplementary Figures 3, 4). On analysis by BC subtypes, estrogen/progesterone receptor (ER/PR)–positive and human epidermal growth factor receptor-2 (HER2/neu)–negative subgroups showed older DNAm age and increased age accel than their counterparts. Similarly, when both the AgeAccelDiff and IEAA were categorized into accelerated age (ACC, positive deviation of DNAm age from age) and decelerated age (DCC, negative deviation of DNAm age from age), the women with ACC had shorter cancer-free intervals than those with DCC in the overall BC and the ER/PR–positive and HER2/neu–negative groups. In the subset within a 5-year follow-up, a 1-year increase in DNAm age was associated with 25% and 40% greater risk for developing BC overall and the ER/PR–positive subtype, respectively (Supplementary Table 4). Similar patterns were observed for age accel in AgeAccelDiff and IEAA in association with BC (Table 3A). However, these trends were less apparent when the entire follow-up period was analyzed (Supplementary Tables 4, 3B).

**Table 3:** Multiple Cox regression for the AgeAccelDiff/IEAA for BC development within 5 years and during the overall period. A. Within 5 years

**< AgeAccelDiff >**			
**BC subtype**	**HR†**	**95% CI**	***P***
Overall	1.18	(0.97, 1.45)	0.103
ER/PR positive	**1.30**	**(1.12, 1.50)**	**0.0004**
**< IEAA >**			
**BC subtype**	**HR†**	**95% CI**	***P***
Overall	1.12	(0.91, 1.39)	0.287
ER/PR positive	**1.18**	**(1.03, 1.36)**	**0.020**
**B. Overall period**			
**< AgeAccelDiff >**			
**BC subtype**	**HR†**	**95% CI**	***P***
Overall	**1.05**	**(1.00, 1.09)**	**0.048**
ER/PR positive	**1.06**	**(1.01, 1.12)**	**0.013**
ER/PR negative	0.97	(0.84, 1.13)	0.702
Her2/neu positive	1.06	(0.90, 1.25)	0.474
Her2/neu negative	1.05	(1.00, 1.11)	0.052
**< IEAA >**			
**BC subtype**	**HR†**	**95% CI**	***P***
Overall	**1.06**	**(1.01, 1.11)**	**0.024**
ER/PR positive	**1.07**	**(1.02, 1.13)**	**0.008**
ER/PR negative	0.96	(0.82, 1.13)	0.647
Her2/neu positive	1.06	(0.88, 1.27)	0.549
Her2/neu negative	**1.06**	**(1.01, 1.13)**	**0.032**

AgeAccelDiff, epigenetic age acceleration measured as departure of DNAmAge from chronologic age; BC, breast cancer; CI, confidence interval; DNAmAge, DNA methylation–based marker of aging; ER/PR, estrogen and progesterone receptor; HER2/neu, human epidermal growth factor receptor 2; HR, hazard ratio; IEAA, intrinsic epigenetic age acceleration as residuals by regressing DNAmAge on chronologic age, adjusted for cell composition. Numbers in bold face are statistically significant.

† HR adjusted by body mass index, waist-to-hip ratio, diabetes at enrollment, healthy eating index-2015, alcohol intake (none, past, < 1 drink/month, < 1 drink/week, 1 to < 7 drinks/week, and 7+ drinks/week), pack-years of smoking (never, < 5 years, 5–20 years, and ≥ 20 years), oophorectomy (none, one and/or part taken out, and both taken out), total duration of unopposed estrogen only use (never, < 5 years, 5–10 years, and 10+ years), and total duration of opposed estrogen plus progestin use (never, < 5 years, 5–10 years, and 10+ years).

### Subset analyses between natural and artificial menopause

**Figure 4:**
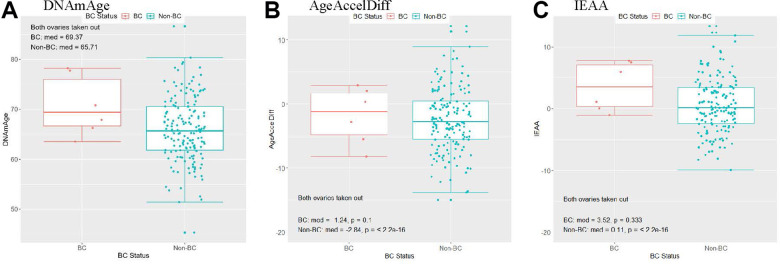
**Women with a history of bilateral oophorectomy: distribution of DNAmAge/AgeAccelDiff/IEAA by BC status.** (AgeAccelDiff, epigenetic age acceleration measured as departure of DNAmAge from chronologic age; IEAA, intrinsic epigenetic age acceleration as residuals adjusted for cell composition; BC, breast cancer; DNAmAge, DNA methylation–based marker of aging). (**A**) DNAmAge, (**B**) AgeAccelDiff, (**C**) IEAA.

Women with natural menopause experienced menarche at a similar age as that in women with bilateral oophorectomy, but the former group experienced menopause at an older age (mean, 50 vs. 43 years, *p* < 2.2e-16), indicating their longer lifetime E exposure. Compared with women having both ovaries intact, women with both ovaries taken out had a greater difference in DNAm age and age accel between the BC and non-BC groups (Figure 4 and Supplementary Figure 5). This corresponds with the results in Table 4: women with artificial menopause had an 80% increase in BC risk by a 1-year increase in DNAm age. Of particular interest, they had a 5-times-higher risk for developing BC for every 10-year increase in age accel in AgeAccelDiff. However, the multiplicative interaction test between oophorectomy and this age accel measure on BC risk was not statistically significant.

**Table 4:** Multiple Cox regression for the DNAmAge/AgeAccelDiff/IEAA for BC development by a history of oophorectomy.

**DNAmAge**			
**Oophorectomy**	**HR†**	**95% CI**	***P***
None	1.00	(0.96, 1.05)	0.823
Bilateral	**1.80**	**(1.56, 2.07)**	**< 0.001**
AgeAccelDiff*§			
**Oophorectomy**	**HR†**	**95% CI**	***P***
None	1.38	(0.88, 2.18)	0.164
Bilateral	**5.08**	**(1.47, 17.51)**	**0.010**
**IEAA**			
**Oophorectomy**	**HR†**	**95% CI**	***P***
None	1.04	(0.99, 1.10)	0.129
Bilateral	**1.37**	**(1.11, 1.69)**	**0.003**

AgeAccelDiff, epigenetic age acceleration measured as departure of DNAmAge from chronologic age; BC, breast cancer; CI, confidence interval; DNAmAge, DNA methylation–based marker of aging; HR, hazard ratio; IEAA, intrinsic epigenetic age acceleration as residuals by regressing DNAmAge on chronologic age, adjusted for cell composition. Numbers in bold face are statistically significant.

†HR adjusted by body mass index, waist-to-hip ratio, diabetes at enrollment, healthy eating index-2015, alcohol intake (none, past, < 1 drink/month, < 1 drink/week, 1 to < 7 drinks/week, and 7+ drinks/week), pack-years of smoking (never, < 5 years, 5–20 years, and ≥ 20 years), total duration of unopposed estrogen only use (never, < 5 years, 5 to 10 years, and 10+ years), and total duration of opposed estrogen plus progestin use (never, < 5 years, 5–10 years, and 10+ years).

*AgeAccelerationDiff as a predictor for BC was analyzed by a 10-year interval.

§A multiplicative interaction scale is reported: Odds Ratio, 1.30 (95% CI: 0.64, 2.66).

Interestingly, the elevated risk for BC by accelerated epigenetic age in women with bilateral oophorectomy remained even after adjustment for BMI and both types of E use. In sensitivity analyses with those with bilateral oophorectomy within BC subtypes (only ER/PR–positive and HER2/neu–negative are available), we confirmed that each subtype group had similar risk magnitude as that of the overall BC group in relation to epigenetic age. Validation tests in general showed coherent directions as those in the discovery tests but with a lack of sample power (Supplementary Table 5 and Supplementary Figure 6).

## DISCUSSION

We investigated the epigenetic age in blood among healthy postmenopausal women by comparing those who prospectively developed BC with those who remained cancer-free. This is of particular interest in the etiology of BC, an age-related disease, and in terms of its primary prevention, considering the deep involvement of DNAm in the aging process, which reflects various molecular alterations at different rates in individuals over time [5–8, 61]. We found that the DNAm age in PBLs tightly correlated with age in the age range studied and that older DNAm age and accelerated epigenetic age were significantly associated with risk for prospective development of BC overall and BC subtypes; these are consistent with those of previous studies [26, 30–36].

BC is a heterogeneous disease characterized by distinct clinical and pathologic features according to different molecular subtypes that undergo unique molecular carcinogenetic mechanisms; thus, examining the performance of epigenetic aging in the BC subtypes is of particular interest. Our findings of older DNAm age and/or increased age accel in both ER/PR–positive and HER2/neu–negative subgroups than in the relevant counterparts and in those without BC development are in line with those of previous tissue-based studies [6, 8, 31, 50, 62]. In particular, estrogen-signaling pathways are key oncogenic drivers of luminal BC, and their activation may have a synergistic effect on breast-tissue aging that promotes cellular proliferation leading to carcinogenesis [8, 39]. Koka et al. [8] supported this hypothesis for the acceleration of tissue aging in the accompanied estrogen signaling process by reporting that DNAm age and age accel were positively correlated with *ESR1* and *PGR* gene expression levels in breast tumor tissue. In contrast, ER/PR–negatives and HER2/neu–positives, typically tumors of more aggressive nature, demonstrated younger DNAm age and decelerated epigenetic aging than their respective counterparts and those without BC; this finding is also consistent with those of prior reports [6, 8, 50]. These non-luminal subtypes may undergo different carcinogenetic processes. The tumorigenic process reflects multiple cellular evolutions, including stepwise, somatic cell mutations, following sub-clonal selection, ultimately forming cancer stem cells with the unique nature of self-renewal and high potential of differentiation and proliferation [63, 64]. Similar to embryonic stem cells, cancer stem cells have a DNAm age close to zero and might play an important role in more aggressive tumors [6]; this explains the decelerated aging observed in our study. These findings support a theory that DNAm age is related to the biologic process of cell differentiation and the maintenance of cellular identity; thus, epigenetic age accel in some way captures intracellular modification in losing cellular identity and changes in cell compositions [65]. Of note, our findings for the ER/PR–positive subtype showed a strong association with DNAm age/age accel when the analysis was restricted to within 5 years, but over the entire period, these correlations became weaker. Thus, we cannot rule out the reverse causality for tumorigenesis of BC to drive the systemic DNAm age in this short follow-up period, calling for further validation studies.

Endogenous steroid hormone pathways and exogenous administration of hormones are both influencing factors for BC development. The mechanism by which these hormones stimulate cellular aging in breast tissues is poorly understood, but it is well hypothesized that estrogen is involved in the development of the mammary gland and epithelial stem cell regulation by regulating cell-cycle progression via the cyclin-dependent kinase pathways and cell proliferation, thus accelerating biologic aging in breast tissues [66, 67]. This aging process begins at puberty and gradually diminishes with advancing age until the last menstrual period [38, 39], so the degree of the effect of hormones on breast tissue aging is reduced after menopause. As evidenced, an earlier age at menarche, a factor for cumulative exposure to estrogen, associated with breast-tissue age accel was observed only in girls [68] and premenopausal women [40]. Of note, the opposite direction (older age at menarche with age accel) is observed in women older than 40 years [69]. Also, a study [22] examining three cohorts of postmenopausal women reported that women with early onset of menopause was epigenetically older, suggesting a causal pathway that faster reproductive aging leads to higher biologic aging [70, 71].

In line with that, bilateral oophorectomy was associated with the increased age accel observed in our and other studies [40]. This supports findings from population and *in vivo* studies [72, 73] that the premature loss of ovarian function before natural menopause increases risk for premature death and age-related diseases. Our study participants with bilateral oophorectomy experienced menopause at younger age than those with both intact ovaries, suggesting shorter lifetime estrogen exposure, and their DNAm age and epigenetic age accel had a substantial impact on the development of BC, whereas those experiencing natural menopause did not show a significant effect of epigenetic aging on BC risk. Bilateral oophorectomy may be accompanied by other synergistic factors for biologic aging that contribute to BC development, such as compromised detoxification, DNA repair systems, and immune surveillance [28].

Obesity, measured via BMI, is one major source of estrogen and an independent BC risk factor in postmenopausal women. Consistent with our study finding, BMI was associated with pan-tissue biologic aging in PB [69] and tissues including breast tissues [8, 40]. Of note, BMI in our study was the only obesity-measured variable that showed greater influence on epigenetic aging in BC than in non-BC groups (a confounder). However, BMI itself did not influence the association of DNAm age with BC development as an effect modifier. Also, while unopposed E users were correlated with older epigenetic aging, opposed E plus P users showed the opposite direction; but neither substantially influenced BC risk through aging acceleration.

Our study focused on whites, and the results should not be extrapolated to other populations. Also, given that data was repurposed from the AS dbGaP repository, samples examined in our study may not fully reflect the source population, with limited confounding information (e.g., variability of hormone therapy) and can result in limited statistical power, particularly for investigating BC subtypes. Our analysis of GEO data did not contain BC subtypes and other reproductive histories, leading to a lack of confirmatory findings; this deserves a future independent, large replication study. Our analysis within a short follow-up period should be interpreted with caution owing to the potential reverse causation. However, our study has potential as the basis for promoting clinical studies to create epigenetically guided decision-making by establishing a comprehensive prediction model for BC risk that better addresses biologic aging processes in BC carcinogenesis. A future study with breast tissues and paired blood from healthy women who are followed with cancer development and prognosis after diagnosis across the menopausal transition with longitudinal epigenetic measures could contribute to understanding epigenetic aging trajectories in the etiology of BC and BC treatment effect by integrating the cumulative hormone effect.

In summary, we found that epigenetically older age and age acceleration led to a greater risk for developing BC overall and ER/PR–positive and HER2/neu–negative subtypes, and these risks were noticeably higher in women with bilateral oophorectomy, independently of their obesity status and exogeneous E use. Our study contributes to the development of a DNAm biomarker that integrates conventional BC risk factors to better reflect the risk for BC subtypes, promoting epigenetically targeted preventive interventions tailored to aged individuals with high risk.

## Supplementary Material

Supplementary Figures

Supplementary Tables
