# Supplementary material for: DNA-methylation age and accelerated epigenetic aging in blood as a tumor marker for predicting breast cancer susceptibility
Source: Aging (Albany NY). 2024 Dec 5;16(22):13534–62. doi: 10.18632/aging.206169 (PMC11723651; doi:10.18632/aging.206169)
Supplement: Supplementary Figures [file aging-16-206169-s001.pdf]

SUPPLEMENTARY FIGURES

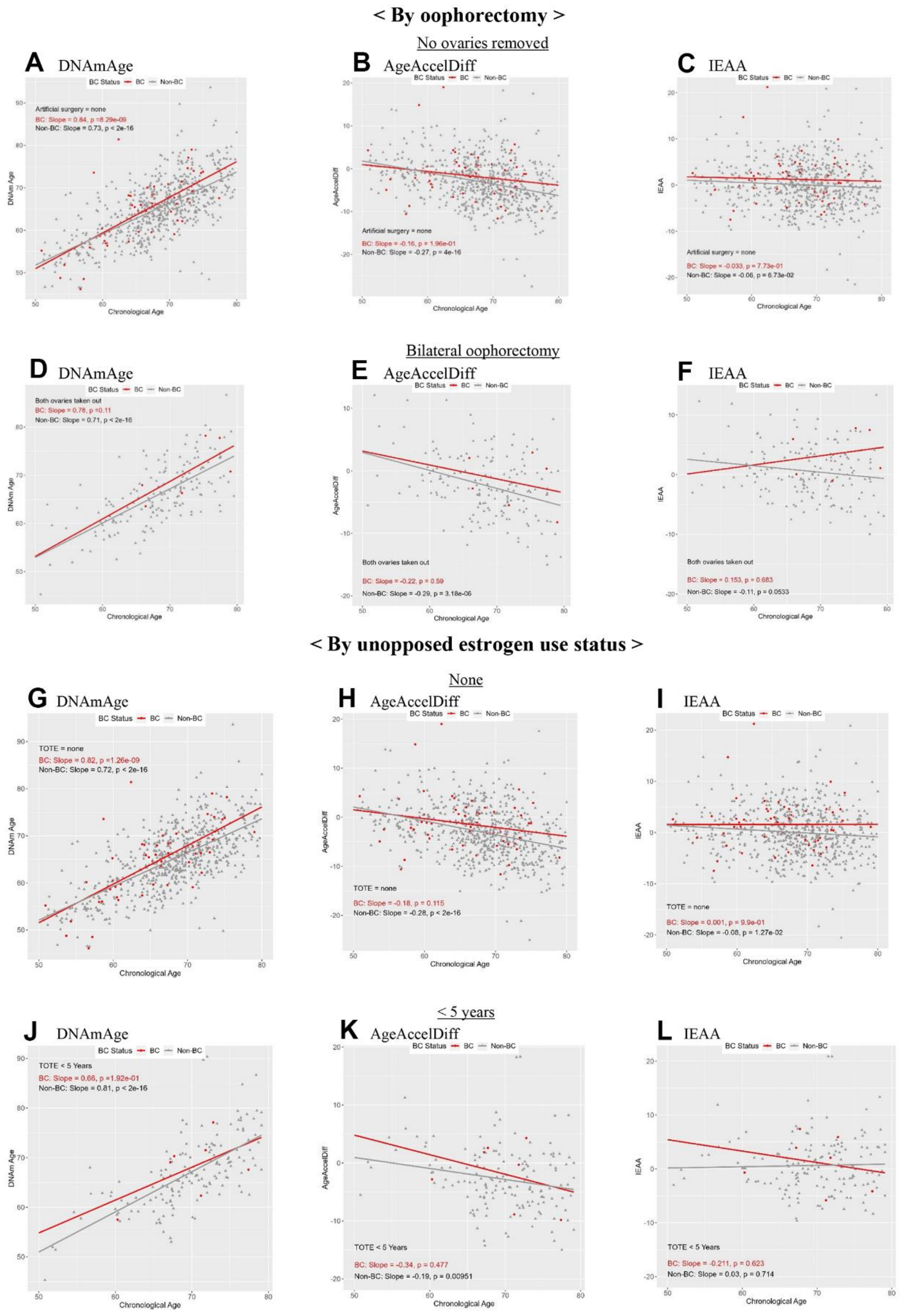

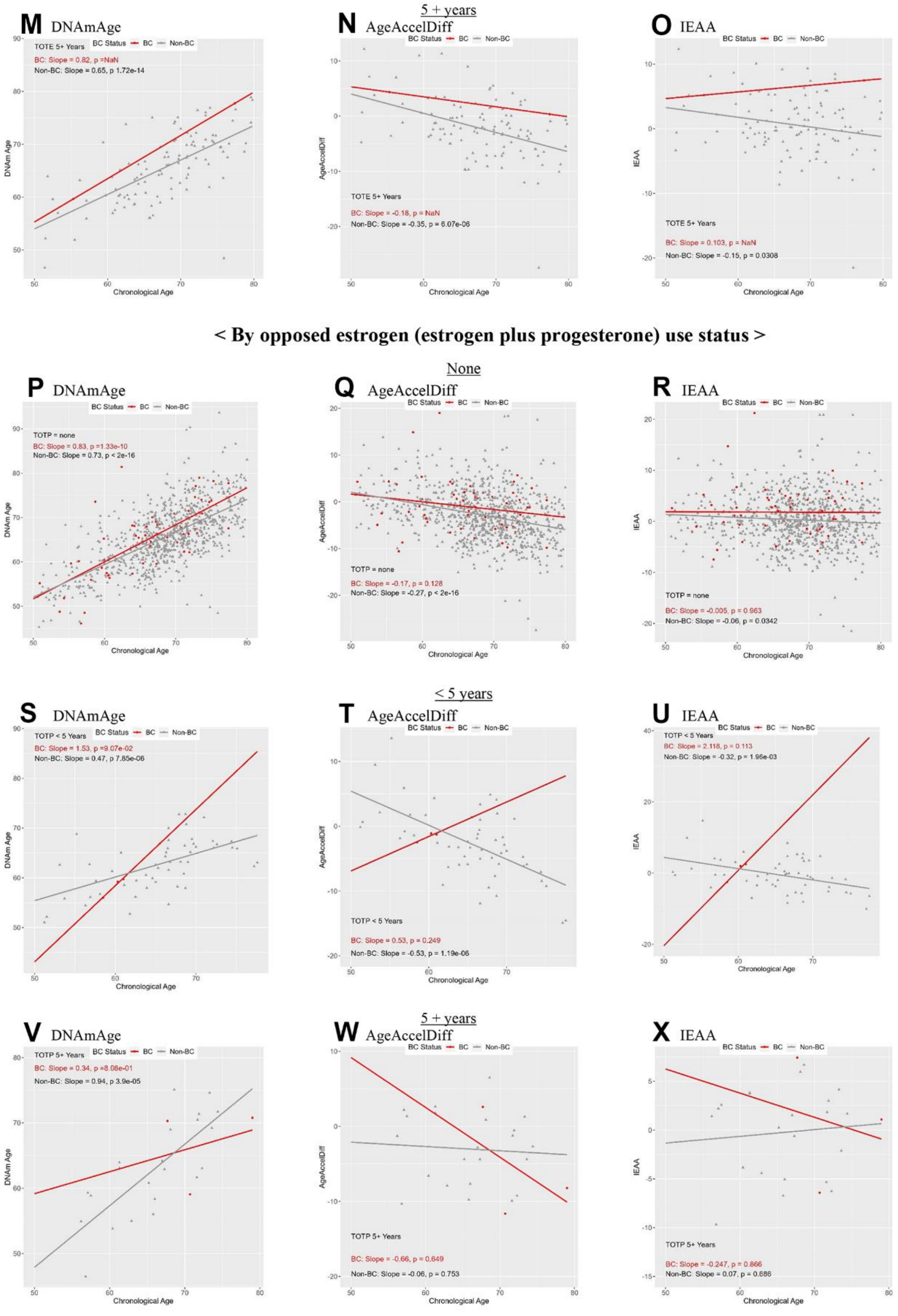

Supplementary Figure 1. Correlation between DNAmAge/AgeAccelDiff/IEAA and chronologic age by BC status. By oophorectomy, (**A**–**C**) no ovaries removed, (**D**–**F**) bilateral. By unopposed estrogen use status, (**G**–**I**) none, (**J**–**L**) < 5 years, (**M**–**O**) 5 + years. By opposed estrogen (estrogen plus progesterone) use status, (**P**–**R**) none, (**S**–**U**) < 5 years, (**V**–**X**) 5 +years. (AgeAccelDiff, epigenetic age acceleration measured as departure of DNAmAge from chronologic age; IEAA, intrinsic epigenetic age acceleration measured as residuals by regressing DNAmAge on chronologic age, adjusted for cell composition; BC, breast cancer; DNAmAge, DNA methylation–based marker of aging).

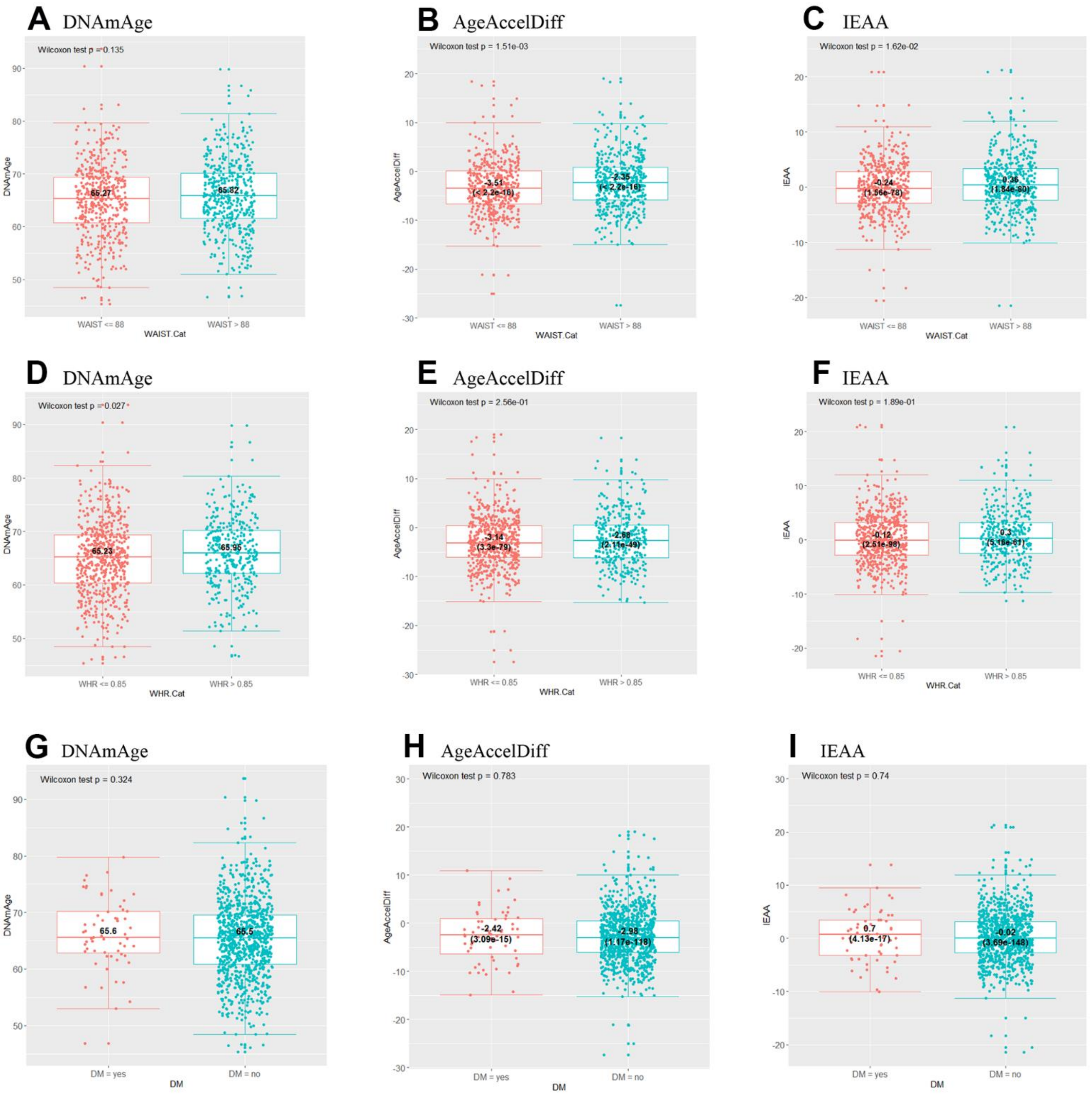
A DNAmAge
Wilcoxon test p = 0.135
65.27
65.82
DNAmAge
WAIST <= 88
WAIST > 88
WAIST.Cat
B AgeAccelDiff
Wilcoxon test p = 1.51e-03
-3.51 (< 2.2e-16)
-2.35 (< 2.2e-16)
AgeAccelDiff
WAIST <= 88
WAIST > 88
WAIST.Cat
C IEAA
Wilcoxon test p = 1.62e-02
-0.24 (1.56e-78)
0.36 (1.84e-80)
IEAA
WAIST <= 88
WAIST > 88
WAIST.Cat
D DNAmAge
Wilcoxon test p = 0.027
65.23
65.95
DNAmAge
WHR <= 0.85
WHR > 0.85
WHR.Cat
E AgeAccelDiff
Wilcoxon test p = 2.56e-01
-3.14 (3.3e-79)
-2.68 (2.11e-49)
AgeAccelDiff
WHR <= 0.85
WHR > 0.85
WHR.Cat
F IEAA
Wilcoxon test p = 1.89e-01
-0.12 (2.51e-98)
0.3 (5.18e-61)
IEAA
WHR <= 0.85
WHR > 0.85
WHR.Cat
G DNAmAge
Wilcoxon test p = 0.324
65.6
65.5
DNAmAge
DM = yes
DM = no
DM
H AgeAccelDiff
Wilcoxon test p = 0.783
-2.42 (3.09e-15)
-2.98 (1.17e-118)
AgeAccelDiff
DM = yes
DM = no
DM
I IEAA
Wilcoxon test p = 0.74
0.7 (4.13e-17)
-0.02 (3.69e-148)
IEAA
DM = yes
DM = no
DM

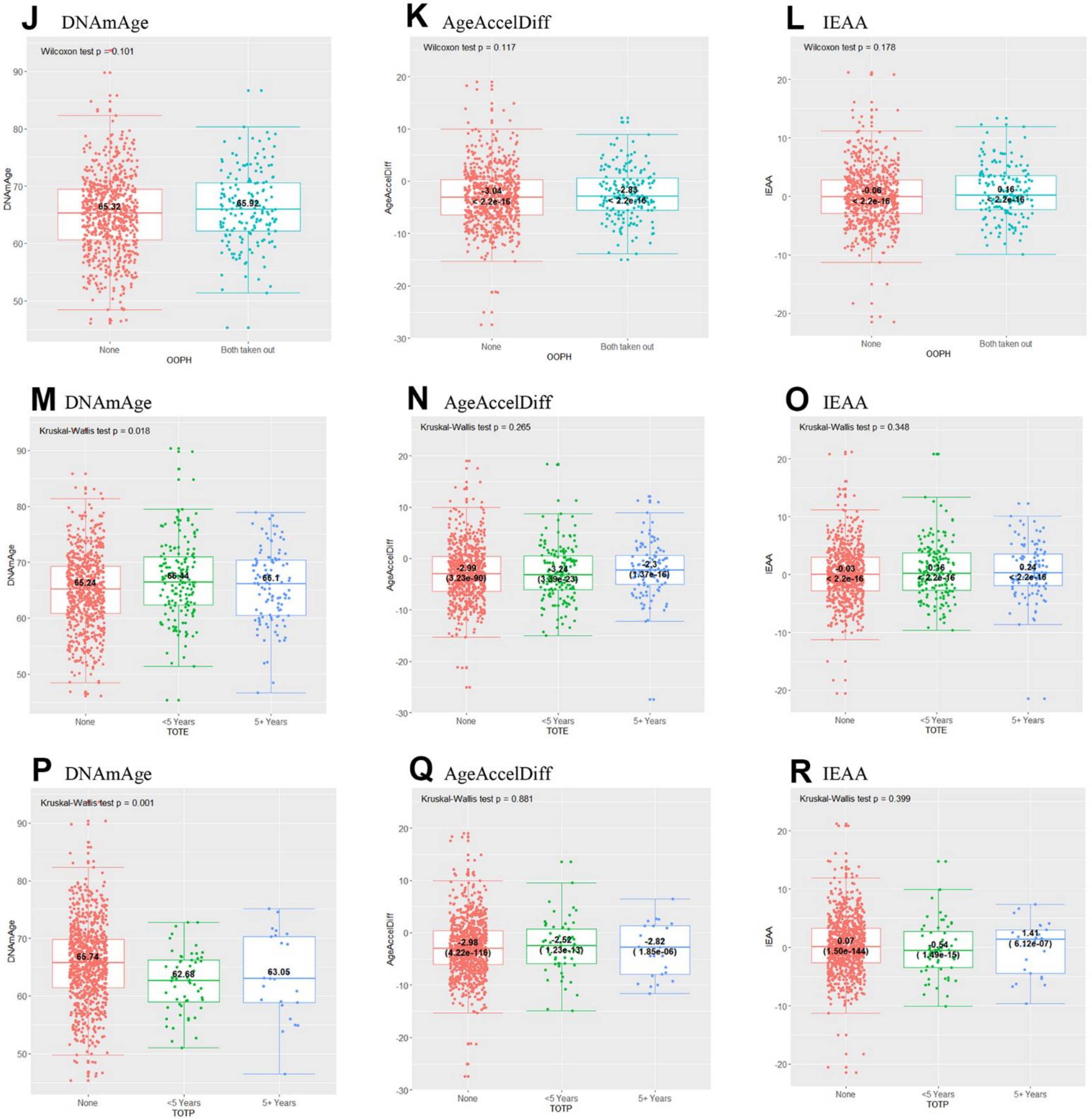

Supplementary Figure 2. Distribution of DNAmAge/AgeAccelDiff/IEAA by BC-risk factors (except body mass index, diet, smoking, and alcohol). By waist circumference, (**A**–**C**); WHR, (**D**–**F**); DM, (**G**–**I**); OOPH, (**J**–**L**); TOTE, (**M**–**O**); TOTP, (**P**–**R**). (AgeAccelDiff, epigenetic age acceleration as departure of DNAmAge from chronologic age; IEAA, intrinsic epigenetic age acceleration as residuals adjusted for cell composition; BC, breast cancer; Cat, Categories; DM, diabetes; DNAmAge, DNA methylation–based marker of aging; OOPH, oophorectomy; TOTE, total duration of unopposed estrogen use; TOTP, total duration of opposed estrogen plus progestin use; WHR, waist-to-hip ratio).

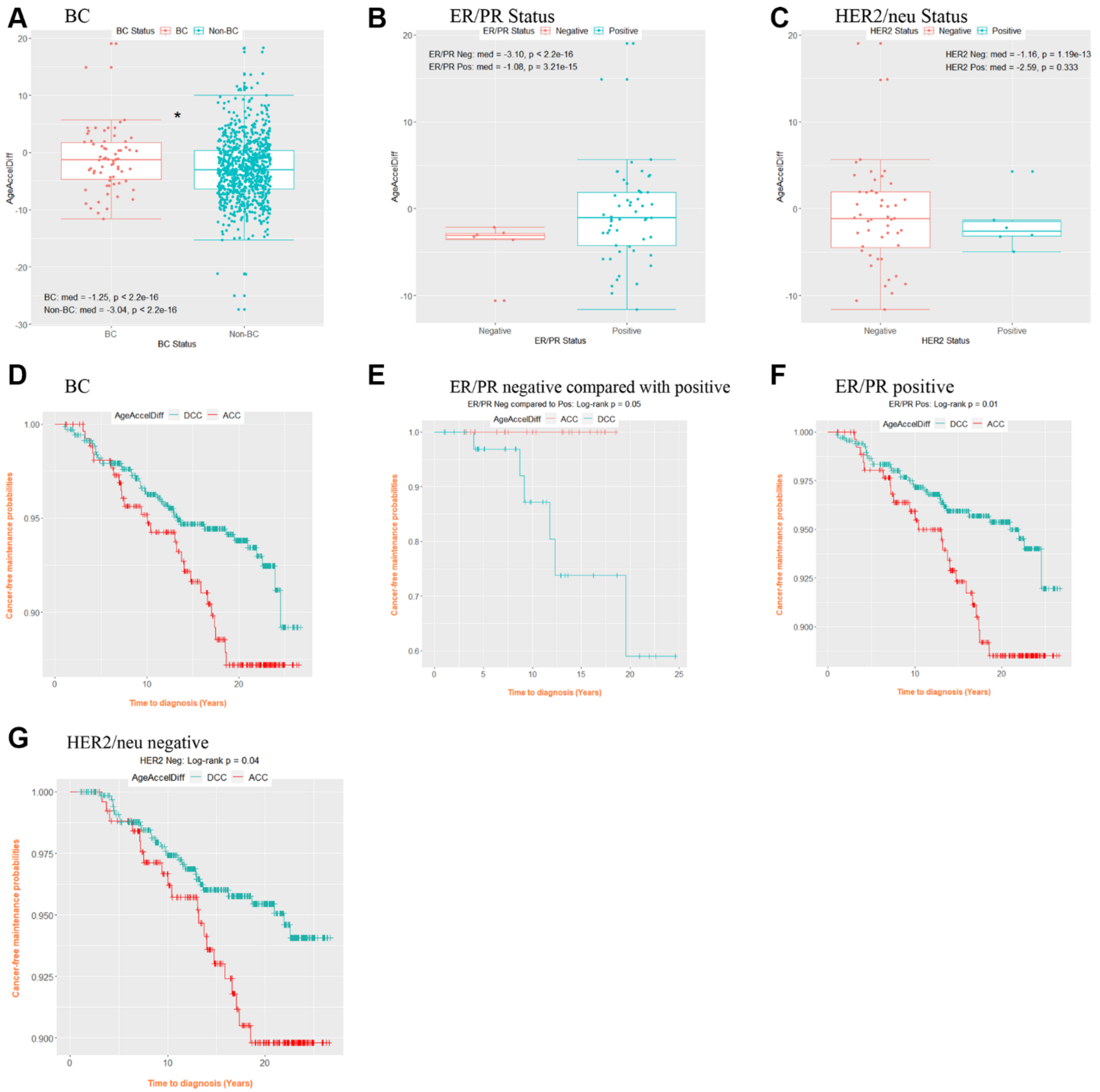

Supplementary Figure 3. Distribution (**A**–**C**) and cancer-free probability curve (**D**–**G**) of AgeAccelDiff by BC status and BC subtype. (AgeAccelDiff, epigenetic age acceleration as departure of DNAmAge from chronologic age; ACC, acceleration, i.e., positive difference of DNAm age from chronologic age; BC, breast cancer; DCC, deceleration, i.e., negative difference of DNAm age from chronologic age; DNAmAge, DNA methylation–based marker of aging; ER/PR, estrogen and progesterone receptor; HER2/neu, human epidermal growth factor receptor 2).

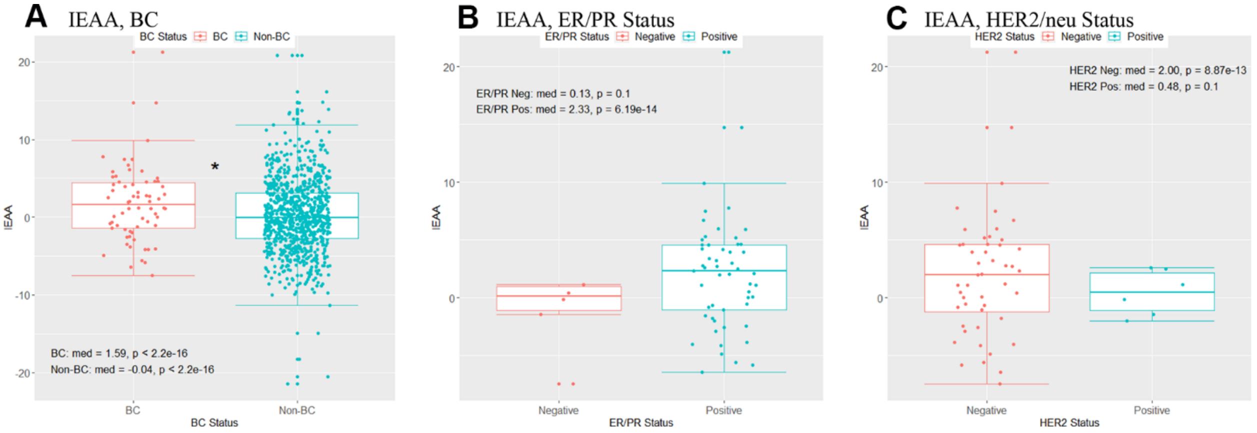

Supplementary Figure 4. Distribution of IEAA by BC status (**A**) and BC subtype (**B**, **C**). (IEAA, intrinsic epigenetic age acceleration as residuals adjusted for cell composition; BC, breast cancer; ER/PR, estrogen and progesterone receptor; HER2/neu, human epidermal growth factor receptor 2).

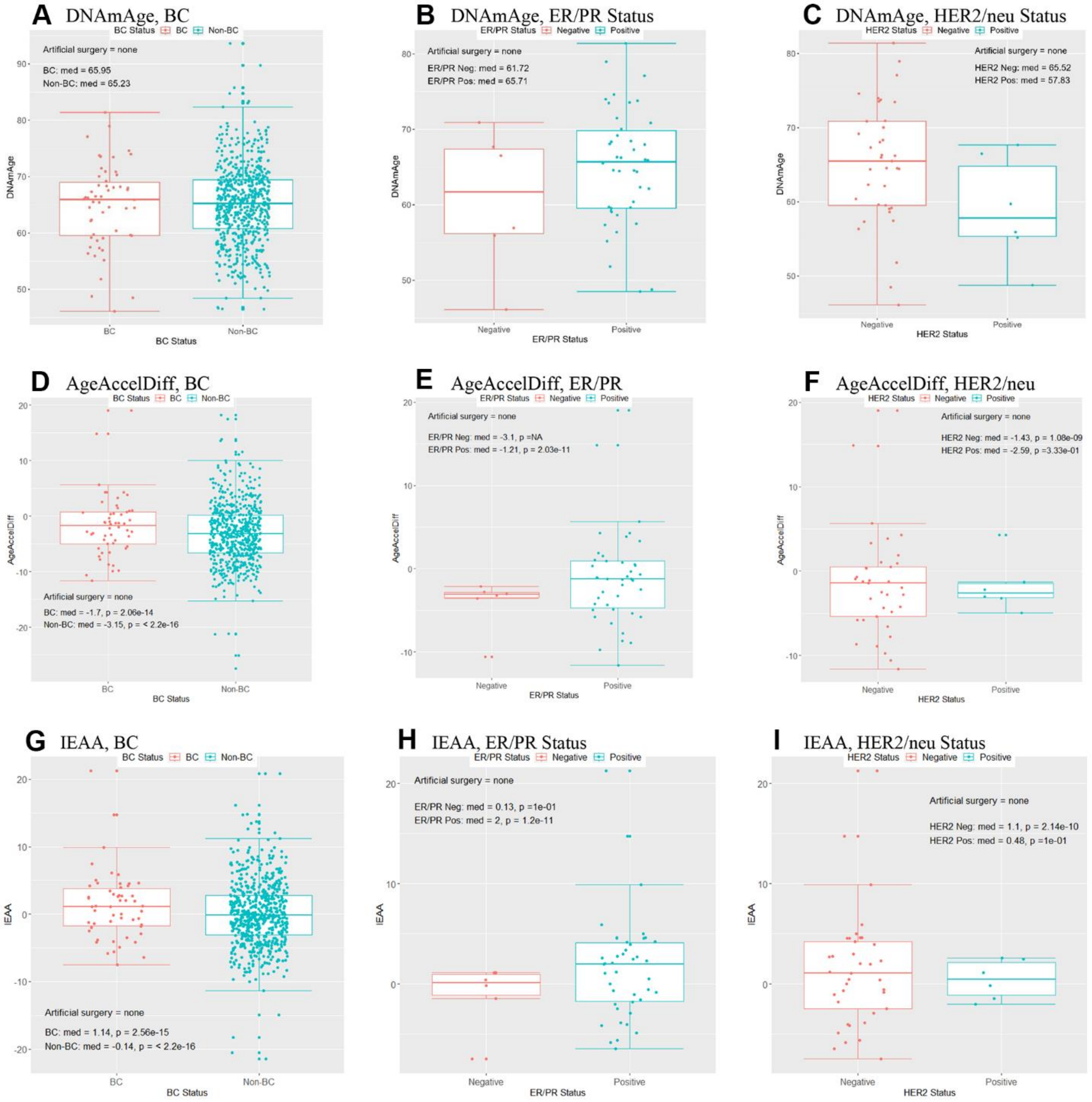

Supplementary Figure 5. Women without a history of oophorectomy. Distribution of DNAmAge (**A**–**C**)/AgeAccelDiff (**D**–**F**)/IEAA (**G**–**I**) by BC status and BC subtype. (AgeAccelDiff, epigenetic age acceleration measured as departure of DNAmAge from chronologic age; IEAA, intrinsic epigenetic age acceleration as residuals adjusted for cell composition; BC, breast cancer; DNAmAge, DNA methylation–based marker of aging; ER/PR, estrogen and progesterone receptor; HER2/neu, human epidermal growth factor receptor 2).

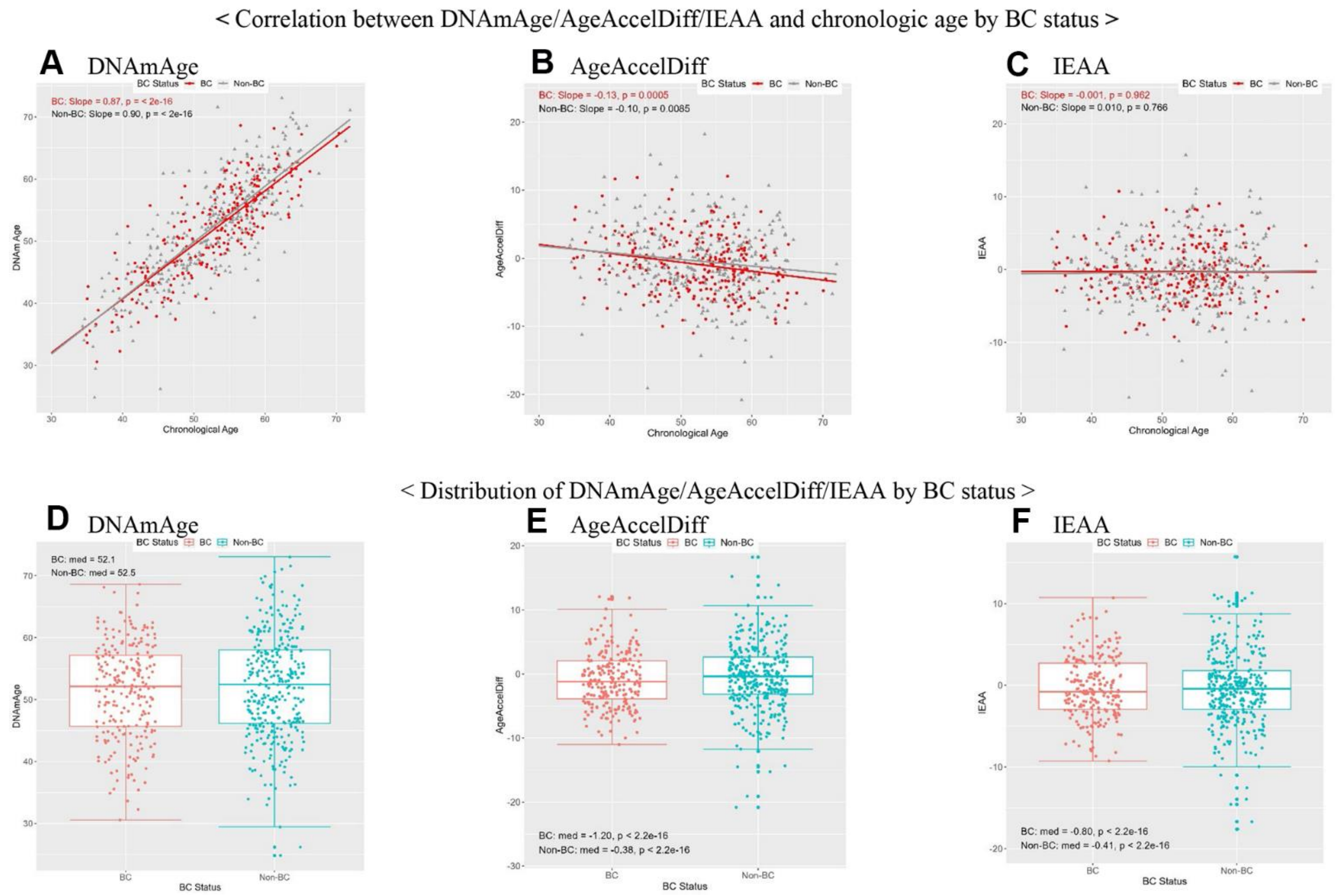

Supplementary Figure 6. GSE51032: validation tests. Correlation between DNAmAge (**A**)/AgeAccelDiff (**B**)/IEAA (**C**) and chronologic age by BC status. Distribution of DNAmAge (**D**)/AgeAccelDiff (**E**)/IEAA (**F**) by BC status. (AgeAccelDiff, epigenetic age acceleration measured as departure of DNAmAge from chronologic age; IEAA, intrinsic epigenetic age acceleration measured as residuals by regressing DNAmAge on chronologic age, adjusted for cell composition; BC, breast cancer; DNAmAge, DNA methylation–based marker of aging).
