# Supplementary material for: DNA-methylation age and accelerated epigenetic aging in blood as a tumor marker for predicting breast cancer susceptibility
Source: Aging (Albany NY). 2024 Dec 5;16(22):13534–62. doi: 10.18632/aging.206169 (PMC11723651; doi:10.18632/aging.206169)
Supplement: Supplementary Tables [file aging-16-206169-s002.pdf]

SUPPLEMENTARY TABLES

Supplementary Table 1. Association of DNAmAge with selected BC-risk factors by BC status*.

A. Among participants with BC development

BC risk factor	Effect size	95% CI	*P*
Age at enrollment	**0.81**	**(0.61, 1.01)**	**2.17E-11**
Body mass index, kg/m^2§ (≥ 18.5 to < 25, normal, vs. ≥ 25 to < 30, overweight)	-1.84	(-6.63, 2.95)	0.445
≥ 30 to < 40, obesity	1.07	(-3.24, 5.38)	0.621
≥ 40, extreme obesity	**16.41**	**(1.65, 31.18)**	**0.030**

B. Among participants without BC development

BC risk factor	Effect size	95% CI	*P*
Age at enrollment	**0.73**	**(0.67, 0.78)**	**5.24E-12**
Waist-to-hip ratio	**7.77**	**(2.05, 13.49)**	**0.008**
Waist-to-hip ratio** (≤ 0.85 vs. > 0.85)	**1.11**	**(0.19, 2.02)**	**0.018**
Healthy eating index-2015	**0.07**	**(0.03, 0.11)**	**0.002**
Healthy eating index-2015¥ (≤ 65.29 vs. > 65.29)	**1.52**	**(0.63, 2.41)**	**0.001**
Pack-years of smoking (never vs. < 5 years)	-0.40	(-1.89, 1.08)	0.594
5 to < 20 years	**-1.61**	**(-3.06, -0.16)**	**0.030**
20 + years	**-1.59**	**(-2.71, -0.47)**	**0.006**
Exogenous estrogen only (never use vs.< 5 years)	**1.89**	**(0.70, 3.07)**	**0.002**
5 to < 10 years	-1.00	(-3.06, 1.06)	0.342
10 + years	1.73	(-0.02, 3.48)	0.053
Exogenous estrogen plus progestin (never use vs. < 5 years)	**-2.94**	**(-4.82, -1.06)**	**0.002**
5 to < 10 years	-2.32	(-6.02, 1.38)	0.219
10 + years	-3.34	(-7.78, 1.09)	0.140

BC, breast cancer; CI, confidence interval; DNAmAge, DNA methylation–based marker of aging. Numbers in bold face are statistically significant.

* Only factors having *statistically significant* association with DNAmAge are displayed.

§ Among BC patients, no one had body mass index < 18.5.

** Waist-to-hip ratio was categorized using 0.85, where cutoff levels or higher fall within visceral obese range [73].

¥ Healthy eating index-2015 variable is dichotomized by a median (= 65.29).

Supplementary Table 2. Association of AgeAccelDiff with selected BC-risk factors by BC status*.

A. Among participants with BC development

BC risk factor	Effect size	95% CI	*P*
Body mass index, kg/m^2§ (≥ 18.5 to < 25, normal, vs. ≥ 25 to < 30, overweight)	0.15	(-3.05, 3.36)	0.923
≥ 30 to < 40, obesity	0.79	(-2.09, 3.67)	0.585
≥ 40, extreme obesity	**21.03**	**(11.17, 30.90)**	**7.04E-05**

B. Among participants without BC development

BC risk factor	Effect size	95% CI	*P*
Age at enrollment	**-0.27**	**(-0.33, -0.22)**	**1.28E-22**
Body mass index, kg/m^2	**0.14**	**(0.08, 0.20)**	**1.28E-05**
Body mass index, kg/m^2 (≥ 18.5 to < 25, normal, vs. < 18.5, underweight)	-0.51	(-3.84, 2.83)	0.766
≥ 25 to < 30, overweight	0.60	(-0.28, 1.49)	0.181
≥ 30 to < 40, obesity	**1.75**	**(0.86, 2.64)**	**0.0001**
≥ 40, extreme obesity	**3.92**	**(1.92, 5.92)**	**0.0001**
Waist circumference, cm	**0.06**	**(0.03, 0.08)**	**6.38E-06**
Waist circumference, cm** (≤ 88 vs. > 88)	**1.09**	**(0.39, 1.79)**	**0.002**
Waist-to-hip ratio	**4.91**	**(0.41, 9.42)**	**0.032**

AgeAccelDiff, epigenetic age acceleration measured as departure of DNAmAge from chronologic age; BC, breast cancer; CI, confidence interval; DNAmAge, DNA methylation–based marker of aging. Numbers in bold face are statistically significant.

* Only factors having *statistically significant* association with DNAmAge are displayed.

§ Among BC patients, no one had body mass index < 18.5.

** Waist-to-hip ratio was categorized using 0.85, where cutoff levels or higher fall within visceral obese range [73].

Supplementary Table 3. Association of IEAA with selected BC-risk factors by BC status*.

A. Among participants with BC development

BC risk factor	Effect size	95% CI	*P*
Body mass index, kg/m^2§ (≥ 18.5 to < 25, normal, vs. ≥ 25 to < 30, overweight)	0.35	(-2.50, 3.20)	0.808
≥ 30 to < 40, obesity	0.54	(-2.02, 3.10)	0.675
≥ 40, extreme obesity	**20.17**	**(11.39, 28.95)**	**2.19E-05**

B. Among participants without BC development

BC risk factor	Effect size	95% CI	*P*
Age at enrollment	**-0.07**	**(-0.12, -0.02)**	**0.011**
Body mass index, kg/m^2	**0.09**	**(0.04, 0.15)**	**0.001**
Body mass index, kg/m^2 (≥ 18.5 to < 25, normal, vs. < 18.5, underweight)	0.25	(-2.76, 3.26)	0.871
≥ 25 to < 30, overweight	0.51	(-0.29, 1.31)	0.211
≥ 30 to < 40, obesity	**1.14**	**(0.34, 1.95)**	**0.006**
≥ 40, extreme obesity	**2.97**	**(1.16, 4.77)**	**0.001**
Waist circumference, cm	**0.04**	**(0.02, 0.06)**	**0.0003**
Waist circumference, cm** (≤ 88 vs. > 88)	**0.78**	**(0.15, 1.41)**	**0.015**
Waist-to-hip ratio	**4.52**	**(0.48, 8.56)**	**0.028**

BC, breast cancer; CI, confidence interval; DNAmAge, DNA methylation–based marker of aging; IEAA, intrinsic epigenetic age acceleration as residuals by regressing DNAmAge on chronologic age, adjusted for cell composition. Numbers in bold face are statistically significant.

* Only factors having *statistically significant* association with DNAmAge are displayed.

§ Among BC patients, no one had body mass index < 18.5.

** Waist circumference was categorized using 88 cm, where cutoff levels or higher fall within visceral obese range [73].

Supplementary Table 4. Multiple Cox regression for the DNAmAge for BC development in different time segments*.

	**Within 5 years**		
**BC subtype**	**HR†**	**95% CI**	***P***
Overall	**1.25**	**(1.05, 1.47)**	**0.010**
ER/PR positive	**1.42**	**(1.29, 1.57)**	< **0.001**
	**Within 5 to 15 years**		
**BC subtype**	**HR†**	**95% CI**	***P***
Overall	**0.94**	**(0.89, 1.00)**	**0.040**
ER/PR positive	0.95	(0.89, 1.01)	0.094
ER/PR negative	**0.83**	**(0.69, 1.00)**	**0.044**
Her2/neu negative	**0.94**	**(0.88, 1.00)**	**0.041**
	**Within 15 years**		
**BC subtype**	**HR†**	**95% CI**	***P***
Overall	0.96	(0.92, 1.00)	0.076
ER/PR positive	0.97	(0.92, 1.01)	0.164
ER/PR negative	**0.84**	**(0.75, 0.95)**	**0.005**
Her2/neu negative	**0.95**	**(0.90, 1.00)**	**0.036**

BC, breast cancer; CI, confidence interval; DNAmAge, DNA methylation–based marker of aging; ER/PR, estrogen and progesterone receptor; HER2/neu, human epidermal growth factor receptor 2; HR, hazard ratio. Numbers in bold face are statistically significant.

* Results from the analyses for the total period are not present owing to no statistical significance.

† HR adjusted by body mass index, waist-to-hip ratio, diabetes at enrollment, healthy eating index-2015, alcohol intake (none, past, < 1 drink/month, < 1 drink/week, 1 to < 7 drinks/week, and 7+ drinks/week), pack-years of smoking (never, < 5 years, 5–20 years, and ≥ 20 years), oophorectomy (none, one and/or part taken out, and both taken out), total duration of unopposed estrogen only use (never, < 5 years, 5–10 years, and 10+ years), and total duration of opposed estrogen plus progestin use (never, < 5 years, 5–10 years, and 10+ years).

Supplementary Table 5. GSE51032: validation tests.

DNAmAge			
**BC**	**OR†**	**95% CI**	***P***
Overall	0.99	(0.97, 1.01)	0.330
**AgeAccelDiff**			
**BC**	**OR†**	**95% CI**	***P***
Overall	0.98	(0.95, 1.01)	0.247
**IEAA**			
**BC**	**OR†**	**95% CI**	***P***
Overall	1.00	(0.96, 1.04)	0.850

AgeAccelDiff, epigenetic age acceleration measured as departure of DNAmAge from chronologic age; BC, breast cancer; CI, confidence interval; DNAmAge, DNA methylation–based marker of aging; IEAA, intrinsic epigenetic age acceleration as residuals by regressing DNAmAge on chronologic age, adjusted for cell composition; OR, odds ratio.

† ORs obtained from univariate analyses.

Logistic regression for the DNAmAge/AgeAccelDiff/IEAA for BC.
